# Indents

**Published:** 1912-04-15

**Authors:** 


### INDENTS.

☞Brief Articles of Technical or Scientific Value will receive notice and comment. Contributions invited.

*Open-Air School.* Mr. Frank. B. Leland, of Detroit, has arranged to give an open-air public school to the city of Detroit, if a site is provided and the board of health and board of education will consent to the work. This school will be used for the instruction of tuberculous children.

*The Public's Estimate of Dentistry.* The heritage of dentistry, that of being considered a mechanical calling, has truly been changed. The hygiene movement has shown to the public that dentistry involves an understanding of pathology and therapeutics. The modern public is informed about bacteria; it knows that all micro-organic life can be divided into two classes, pathogenic and non-pathogenic; that the one great thought in regard to the art of healing is prevention and not cure; that a reasonable amount of the physical ills that humanity is heir to is preventable; that dentistry is part and parcel of the healing art, and as such owes its services to humanity in regard to prevention. In other words, the tooth carpenter has been displaced by the tooth doctor.

The methods and practices of the average dentist will have to change in the future because of this knowledge. The patient, on presentation for service in the oral cavity will be examined not in regard to teeth with cavities to be filled or to spaces to be filled by artificial substitutes; his oral condition will be considered from the standpoint of pathology, requiring a diagnosis and also a prognosis. In other words the dentist will have to endeavor to find a cause and remove it before dismissing his patients.—Dr. Flanagan, in *Cosmos.*

*Conditions of Tongue and Buccal Cavity: What May be Learned from them.*

Glossitis is not frequently encountered in children, but occasionally we find excoriations and patches covered with macerated epithelium which persist without any visible cause. In measles, variola, erysipelas, herpes, pemphigus, and eczema, eruptions may appear on the tongue; later the bullae or vesicles may ulcerate.

A tongue with a yellow coating on its base indicates hepatic torpidity, and is called by many physicians the "podophyllin tongue."

A dry glazed tongue usually indicates gastric or enteric disease, although it is also noted in phthisis and wasting disorders. Gastritis, enteritis, peritonitis, and intestinal obstruction present this peculiar dry, glazed tongue.

The "strawberry tongue" is an evidence of scarlatinal infection or of ichthyosis linguae. (Differentiation is easy, of course.)

A smooth, moist, red tongue generally exists in hyperacidity.

A fissured tongue is seen in diabetes, syphilis, chronic dysentary (with peculiar glazing), and in marked cases of erysipelas.

A red, swollen tongue will be noted in aneurism of the aorta, cretinism, mitral disease, variola, and pemphigus. Glossitis, carcinoma of tongue, and ingestion of irritant poisons will also cause this appearance. Some cancerous patients present a "large red tongue."

A small tongue is seen in bulbar paralysis (the patient may be unable to protrude the tongue or only one side of it), the typhic state, acute peritonitis (red and glazed), enteric fever, chronic gastritis.

A large, pale tongue, showing indentations of teeth, may be seen in anemia, gastric atony, neurasthenia, mucous disease (it is slimy), cancer and ulcer of stomach, relapsing fever, and chronic gastritis in anemic, neurasthenic patients.

A dry, furred tongue is present in ague, dyspepsia, continued fevers, the exanthemata, erysipelas, jaundice, pyemia, pneumonia, typhus, acute tuberculosis, endocarditis, lead poisoning, remittent fever.

White fur is more or less marked in alcoholism, apoplexy, hepatic disease (catarrh, gastric, biliary or enteric), colitis constipation, erysipelas, enteric fever (down the center, and early), measles, meningitis, rheumatism, acute pneumonia, lithemia, quinsy, gout, phthisis, relapsing fever, gastric irritation, migraine.

A brown fur is seen in severe erysipelas, third week of typhoid fever, gout, gastritis, remittent fever, jaundice, septicemia, scurvy, strangulated hernia, typhus, typhoid condition, acute tuberculosis. Any dirty tongue may assume a brown tint from coffee, cocoa, licorice, etc.

Ulcers on the tongue may mean aphthae, chancre, gastritis, épithelioma or a rough tooth; secondary and tertiary syphilis cause patches and ulcers. Ulcers on the under side of the tongue suggest sprue.

Nodules point to actinomycosis.

A bitten tongue leads us to suspect epilepsy or hystero-epilepsy (not hysteria); a fall may cause the tongue to be severely bitten.

The tongue trembles in alcoholism, bromism, chorea, bulbar paralysis, delirium tremens, paralysis (general) disseminated sclerosis, the typhoid condition, and often just before death.

(The foregoing is reproduced from that excellent little, book, by Dr. G. H. Candler, on "Everyday Diseases of Children." The portion here quoted is of unusual value to the busy family doctor, deserving to be closely scrutinized and the facts stored away in a memory's niche for future—and frequent—reference; for here Dr. Candler has collated and grouped concisely and succinctly all the salient facts connected with the more frequent lesions observed in the buccal cavity as valuable diagnostic factors. There is danger that among the younger generation of the guild these and similar diagnostic helps in the treatment of disease are being slighted and forgotten—to the detriment of the patient and the prejudice of the profession.)—*Am. Journal Clinical Medicine.*

*Seal the Remedy.* In connection with the use of formocresol I want to take advantage of this opportunity to emphasize one point which I am confident is overlooked by many dentists who are daily using this remedy and who are getting fairly good results, but not the best of which the remedy is capable of producing. Formocresol is indicated wherever putrescence is present in a canal or where the formaldehyde gas can be prevented from coming in contact with the live tissue in the periapical area. The point I wish to emphasize, if possibly more forcibly than I have ever done before, is that the remedy, whenever used, *should always be sealed in the tooth with cement.* So-called temporary cements are now on the market and, to my mind, they fill a long-felt want. I am certain that the best results will be obtained in the use of any remedy when we follow the general rule that all remedies should be sealed with cement. This will force us to correctly diagnose the condition and employ the means and remedies indicated in the rational treatment of the case. Until we are willing to spend the necessary time to accomplish this end, we shall not be living up to the highest ideals of our calling. I know that remedies can be hermetically sealed in teeth with gutta-percha or some of the temporary stoppings, and many use the latter plan for sealing purposes in preference to cement for the reason that, should the tooth ache, the dressing is easily removed by the operator or by the patient at home; and the giving of instruction along this line is a daily occurrence in many dental offices. He who uses temporary stopping because it is easily removed, and instructs his patient how to remove it should the tooth ache, either does not understand the condition he is treating or else he has no confidence in his remedy. If the case has been correctly diagnosed and the proper remedy has been hermetically sealed in the cavity without pressure, the tooth will not ache for any length of time—it matters not what the pathologic condition may be.—J. P. Buckley, *Dental Review*.

*Sterilizing Instruments.* The use of alcohol as a means of sterilization is rapidly increasing. Surgical instruments, which formerly were boiled, are placed in 75 to 95 per cent. alcohol and are found to be completely aseptic upon their removal.

This fact should be of interest to the dental profession. It is almost impossible to completely dry certain dental instruments, such as broaches, burs and hypodermic needles, after boiling them in any aqueous solution, and consequent rusting is sure to result. In this condition they are positively dangerous. But if they are cleansed of any debris with a dry brush and then placed in a jar of alcohol they will be rendered sterile with no danger of rusting or any loss of temper. About ten minutes is all that is necessary to allow them to remain, but they can be left there indefinitely without harm.

A covered flat glass jar of sufficient size to contain the larger instruments can be procured at slight expense, and as the instruments are used they can be placed in this, thus sterilizing them several times during an operation, something which cannot be done by the boiling process because of the time involved. It will save the cost of an electric or gas sterilizer, which is apt to be considerable; save the wear on the instruments and give absolute results, which is all that can be desired.—Edward L. Wharton, in *Scrap Book*.

*Essentials in Business.* Recently a representative from a school of salesmanship in an address before an association of merchants, said:

"Back of everything you sell is your character, and when your community believes that, you will find your customer, gentlemen, and you will keep him."

"The best advertisement of your business is yourself."

"The only way you can get the confidence of a customer is by treating him absolutely square."

The speaker laid down four essentials of good business, "honesty and truthfulness of statement, pertinency, timeliness and persistency."

How applicable all of this is to the dentist. One of the greatest essentials is a good character. A man whose character is above reproach will command the greatest respect from his patients. And the dentist who is strictly honest with his patients, and in his operations, striving every time to give value received, will be well thought of.

A pleased patient is sure to speak well of you to others. —*Digest.*

*Bridges.* The cantelever bridge offers us some valuable lessons in constructing our saddles. We all have seen these bridges stand for years, giving good service and remaining in their proper position. Of course, I mean the cantelevers in the bicuspid and molar regions, and also the dummy having a full occlusal surface. But we have about discarded these bridges because of the great number of failures. If the cantelever dummy had been constructed as we now construct our dummies, resting upon the ridge with some pressure, do you think this would lessen the number of failures? No doubt this would help, but it would not solve the problem entirely. We would find the dummy resting too heavily upon the ridge and finally be imbedded in the gum. The lesson to be learned is that the ridge, covered to offer sufficient support, must be greater than the occlusal surface of the dummy. This, with the fact, calling for ample support from the abutments, gives us a real and broad field for working out many satisfactory appliances.

All saddle-bridges must have a close-fitting saddle, even distribution of pressure on the ridge, proper and sufficient anchorage, the teeth borne by the saddle arranged as directly over the ridge as the case permits, and an intelligent understanding of the appliance by the patient. All persons wearing these bridges certainly value them more than a plate, and thus you are aided in giving more time and thought to its construction and care.

The fixed saddles have many advantages over any other appliance, ease in cleaning, not demanding any more care or attention than the adjoining teeth; freedom from annoyance of removal to cleanse, ease of mind, knowing it will not loosen or be misplaced, and a feeling of permanency and security.—Dr. Kellogg, in *Summary*.

*New Electrical Discovery.* "Almost by accident," said Professor M. I. Pupin, of Columbia University, "I have discovered a device which magnifies an electric current. I applied only a feeble current to my first machine and it was broken to pieces by the increased power derived."

Professor Pupin made this statement at a meeting of the National Academy of Science, held in the new library of the university, New York. He went on to say that with his new invention he could drive a train with the current used in the telephone and could take a weak wireless current and make it strong enough to be easily read. He said he was certain the principle was heretofore unknown, and that he would go into details at the next meeting of the academy in April.—*Scrap Book.*

*Repairing Vulcanite Dentures.* It is of interest to all dentists who in their practice sometimes have occasion to repair Vulcanite Dentures, that a perfect union can be obtained of old and new rubber by a very simple method, having been tested on a highly polished surface of old rubber, to the satisfaction of the most skeptical. Using the ordinary method of flasking a case (and omitting the usual preparation of retaining cuts) by coating the surfaces for new attachment with slight covering of crude petroleum, the ordinary pressure caused while being vulcanized will cause the union of rubbers to become inseparable. This process is of value in many instances of repair work, in strengthening a weak and thin plate, additional portions to plates, dry packing, etc. Try a case for your own satisfaction and you will be convinced of its value.—C. W. F. Holbrook, in *Scrap Book.*

*Formula for Tooth Paste.*

| | |
|---|---|
| Mass solution | 200 c.c. (mils). |
| Precipitated chalk | 500 grammes. |

Mix, and put into collapsible tubes at once.
The tubes should stand a day or two before use.

MASS SOLUTION.

| | |
|---|---|
| Gelatin, in small pieces | 30 grammes. |
| Castile soap (moist) | 60 grammes. |
| Saccharin | 8 grammes. |
| Menthol | 8 grammes. |
| Oil of eucalyptus | 8 c.c. (mils). |
| Oil of wintergreen | 22 c.c. (mils). |
| Glycerin | 1000 c.c. (mils). |
| Water | 500 c.c. (mils). |
| Hot water | 500 c.c. (mils). |

Soak the gelatin in the water over night; dissolve the soap and saccharin in the hot water; mix the menthol, oils and glycerin. Pour all together in the order named, and let the mixture stand a day or two before placing in collapsible tubes.—*Druggists' Circular.*

*Incidents of Office Practice.* The building in which my office is located has reversible sash frames, the windows are cleaned from the inside, some force is required to reverse the frames and a loud, creaking, ripping sound comes out each time it is done. A fifteen-year old boy and his mother were in the reception room awaiting the boy's turn for an extraction. I was working in the operating room and the window cleaner was in another room. Just as my patient awoke from the gas he yelled and at the same time the window cleaner reversed a sash with the usual terrifying noise. The boy made for the front door, yelling to his mother, "Come on, ma, no tooth out for me when it sounds like that coming out."

An Irish girl wanted several teeth extracted, and, pointing to an upper bicuspid, she said, "Above all things dear doctor take out this wan, it 'leps'."—Dr. "Mapp of Brooklyn" in *Scrap Book*.

*Professional Attitude.* Speaking to a nurse who spends much of her time in a metropolitan maternity ward she casually remarked, "the visiting surgeon of the ward has such love for young children that frequently he has taken the new born child in his arms and walked up and down the floor talking animately to the child, his face the while aglow with a smile of delight." So great was his interest in his work he never has shown irritation or lack of sweetness of dispostion under the most trying circumstances.

Impressed by the narration, I am led to believe this may be the key to a successful prosecution of dental practice.

Difficult cases are made easier, provoking situations less dreadful, obstinate conditions more yielding and a greater pliability of patients is obtained where the operator is so master of himself that a sweetness of temperament is maintained throughout all his work.

Such an attitude permits the dentist to render service far superior to anything he has before achieved and will win for him, not alone the commendations of grateful patients, but the love and esteem of every one with whom he comes in contact, and will become reflected in a countenance indication of a high type of manhood.—Dr. Gregory in *Scrap Book*.

*A Question of Ethics.* Is not the direct seeking of patronage plainly forbidden by the letter and spirit of our ethics? We think that this is not only so, but that it constitutes one of the cardinal principles of our professionalism. Done by request or specific understanding it redeems a promises—it discharges a duty —it serves a splendid purpose and is within the bounds of ethics.

Done in the absence of either it is in effect, a seeking of patronage, and is not, in the writer's opinion, within the bounds. Truly, the question is largely academic, but its discussion at this time will be opportune, indeed. Personal opinion cannot be entirely eliminated from its discussion. —J. H. Crossland.

